# Variable effects of local management on coral defenses against a thermally regulated bleaching pathogen

**DOI:** 10.1126/sciadv.aay1048

**Published:** 2019-10-02

**Authors:** Deanna S. Beatty, Jinu Mathew Valayil, Cody S. Clements, Kim B. Ritchie, Frank J. Stewart, Mark E. Hay

**Affiliations:** 1 School of Biological Sciences and Aquatic Chemical Ecology Center, Georgia Institute of Technology, 311 Ferst Dr., Atlanta, GA 30332, USA.; 2 Department of Natural Sciences, University of South Carolina Beaufort, 801 Carteret St., Beaufort, SC 29902, USA.

## Abstract

Bleaching and disease are decimating coral reefs especially when warming promotes bleaching pathogens, such as *Vibrio coralliilyticus.* We demonstrate that sterilized washes from three common corals suppress *V. coralliilyticus* but that this defense is compromised when assays are run at higher temperatures. For a coral within the ecologically critical genus *Acropora*, inhibition was 75 to 154% greater among colonies from coral-dominated marine protected areas versus adjacent fished areas that were macroalgae-dominated. *Acropora* microbiomes were more variable within fished areas, suggesting that reef degradation may also perturb coral microbial communities. Defenses of a robust poritid coral and a weedy pocilloporid coral were not affected by reef degradation, and microbiomes were unaltered for these species. For some ecologically critical, but bleaching-susceptible, corals such as *Acropora*, local management to improve reef state may bolster coral resistance to global change, such as bacteria-induced coral bleaching during warming events.

## Teaser

Local management enhances a coral’s defense against a thermal-bleaching pathogen.

## INTRODUCTION

Coral reefs are biodiverse and productive ecosystems upon which coastal societies rely for ecosystem services such as protection from storm surge, tourism-generated revenue, and food security (*1*). However, reefs are declining worldwide, notably from a fivefold increase in the frequency of thermal stress events in recent decades (*2*). Consequences of these heating events can be exacerbated by other stressors, including disease, ocean acidification, pollution, and increases of harmful macroalgae following removal of herbivorous fishes (*3*, *4*). These interacting stressors can harm or kill corals through diverse mechanisms, including alterations in how corals interact with microorganisms (*5*–*7*). Determining how declines in coral and increases in macroalgae interact with other stressors, such as ocean warming and disease, is challenging but may be vital for understanding and mitigating losses of corals in a changing ocean.

Evidence suggests that coral health is affected by interactions with microbial associates. As examples, bacterial mutualists can help corals obtain nutrients and may also suppress or consume coral pathogens (*5*, *8*, *9*). Coral bleaching and tissue loss due to pathogens, such as bacteria of the genus *Vibrio*, increase when waters warm, in part, because of conditions that promote *Vibrio* growth and virulence (*7*, *10*, *11*). However, temperature-induced coral loss may also occur because of shifts in the beneficial bacteriome, allowing for *Vibrio* colonization and proliferation within the coral microbiome (*8*, *9*). The relative contribution of direct thermal stress versus bacterial-induced bleaching (*11*) or other mechanisms, such as hyperoxic stress (*8*), that disrupt the symbiotic relationship between coral and their photosynthetic microalgal partners is not fully understood but postulated to be important (*8*, *11*–*13*). As evidence of this importance, corals regulate their microbiomes via production of antimicrobial peptides, sloughing of mucus, and direct consumption of microbes or by hosting predatory microbes or phages that attack microbial pathogens (*6*, *9*). As thermal stress events become more frequent and algal cover increases (*2*, *4*), corals may lose the ability to regulate their microbiomes (*7*–*9*) and may lose defenses produced by commensal microbes (*5*).

Microbial dysbiosis (the loss of beneficial, or increase of harmful, microbes) is reported from reefs with abundant macroalgae (*14*). Macroalgae are suggested to disrupt coral microbiomes via transfer of allelochemicals (*9*) or microbes (*15*, *16*) or release of dissolved organic carbon that affects microbial growth (*17*). Bacterial density and virulence gene abundance are enriched in the benthic water within ~25 cm of the reef surface on algae-dominated compared to coral-dominated reefs (*18*). Furthermore, microbiomes of corals from algae-dominated sites can be more variable than those of corals from coral-dominated sites (*19*, *20*). Greater variability of host microbiomes among stressed individuals appears pervasive across a range of ecosystems and species and may be indicative of another form of microbial dysbiosis [the Anna Karenina principle; (*21*)]. These patterns suggest that abundant macroalgae, like high temperature, may impair a coral’s ability to regulate its microbiome (*19*, *21*) and possibly the ability of the coral, or its microbiome, to control pathogen adhesion and persistence (*6*, *9*).

Although coral defenses against pathogens could be produced by the coral host, its symbiotic microalgae, or other components of its microbiome (*6*, *9*, *22*), discovery of antibiotics from hard coral hosts appears rare compared to soft corals or other marine invertebrates (*23*, *24*). In contrast, microbial associates of hard corals, like those of other invertebrates, commonly produce antimicrobials (*6*, *8*, *9*). Thus, bacterial associates of hard corals are a reasonable group to explore for their potential role in generating anti-pathogen defenses. However, the relative effect that local and global stressors may play in influencing coral anti-pathogen defenses, whether host- or microbially produced, is not well understood.

Evidence suggests that marine protected areas (MPAs) can restore critical ecosystem processes and function to reefs, for example, by promoting greater coral recruitment and cover following enhanced macroalgal removal by herbivorous fishes (*25*–*28*). Some MPAs also have lower levels of disease due to reductions in coral damage from derelict fishing lines (*29*). We sought to test the hypothesis that no-take MPAs (fishing prohibited) promote coral defenses toward a coral-bleaching pathogen. We used three sets of paired reefs that differed in benthic community composition as a result of 10 to 12 years of local management before our study. During that period, fishing was prohibited, herbivory increased by three- to sixfold, and macroalgae reduced by 75 to 95% in protected versus fished areas (*27*, *28*, *30*).

To assess coral anti-pathogen defenses, we selected three common coral species (*Acropora millepora*, *Porites cylindrica*, and *Pocillopora damicornis*) that differ in their life history traits and susceptibility to bleaching. To test for coral defenses, we shook 50-ml volumes of 10 separated individuals of each coral in 50 ml of local seawater for 20 s, froze the water from this activity (hereafter called “coral water;” see Methods), freeze-dried and ultraviolet C (UV-C)–irradiated the water to kill all associated microbes, and tested the ability of this water to suppress the common coral pathogen *Vibrio coralliilyticus* (BAA-450). We tested for coral anti-pathogen defense using laboratory bioassays conducted at 24° and 28°C. We chose *V. coralliilyticus* as an ecologically realistic assay pathogen because it targets diverse coral groups (agariciids, acroporids, and pocilloporids), is distributed worldwide (*10*), becomes virulent under elevated temperatures (*10*, *31*), and causes coral bleaching and mortality throughout the Indo-Pacific (*13*, *31*, *32*). Moreover, this bacterium is congeneric with other species that cause coral bleaching (*13*) and therefore may be useful for exploring coral response to temperature-activated pathogens in general. In addition, we assessed whether the potency of each coral’s anti-pathogen defense differed between three pairs of coral-dominated MPAs and adjacent macroalgae-dominated areas (fishing allowed) scattered along 11 km of coastline on the southern coast of Viti Levu, Fiji. We also evaluated whether changes in reef state (MPA versus fished) and coral anti-pathogen defense correlated with changes in coral microbiome variability [as suggested by the Anna Karenina principle (*21*)].

On our study reefs, coral and algal cover differed markedly between adjacent fished and MPA areas because of differences in the abundance of herbivorous fishes (*28*, *30*), and these fished and MPA areas were replicated (*n* = 3) and interspersed. This framework represented a decade-long manipulation conducted by Fijian villagers where treatment reefs (MPAs) were protected from fishing and immediately adjacent control reefs were fished. This facilitated tests of the effects of protection (producing coral-dominated versus macroalgae-dominated reefs) on coral defenses and microbiomes in the absence of confounding physical variables across sites. We found that the inhibitory effects of coral water against *V. coralliilyticus* differed significantly on the basis of temperature, coral species, and inoculum density of the pathogen. We also found that *A. millepora* [falling within one of the most abundant, diverse, and ecologically important genera of reef-building corals (*33*–*35*)] had enhanced anti-pathogen defense if it occurred in coral-dominated MPAs versus macroalgae-dominated fished areas. Differences in reef state and anti-pathogen defense of *A. millepora* coincided with differences in key indicator bacterial strains and with changes in microbiome variability. Anti-pathogen defenses of *P. cylindrica* and *P. damicornis* did not differ as a function of reef state, nor did the composition or variance of their microbiomes. We discuss findings for *Acropora* as potentially linked to subtle changes in the beneficial microbiome or to microbiome-independent changes in the coral.

## RESULTS

**Fig. 1:**
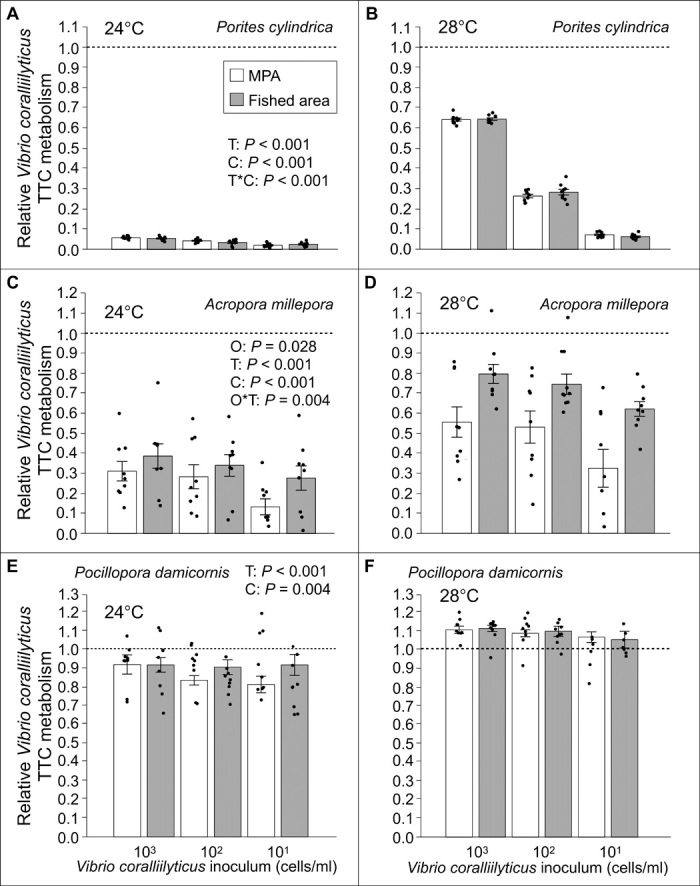
Effects of coral water on *Vibrio coralliilyticus*. Mean (±SE) activity of coral water relative to reef water against *V. coralliilyticus* (quantified by metabolism of tetrazolium chloride) at 24° and 28°C for *P. cylindrica* (**A** and **B**), *A. millepora* (**C** and **D**), and *P. damicornis* (**E** and **F**). The dashed line at 1.0 is the expected value if there is no effect. Values below this line indicate suppression of *V. coralliilyticus* metabolism, and values above the line indicate enhancement. *P* values are from linear mixed-effects models implemented with Akaike information criterion for model selection (*n* = 9). Factors remaining for each species’ model after the model selection process are provided. O, T, and C represent origin (MPAs or fished areas), temperature, and concentration of pathogen inoculum, respectively. Dots indicate individual data points. One data point with negative values (after reduction of the optical density of sterilized coral water; see Methods for information on data processing) is not depicted in each of (C) (data point value is −0.040) and in (D) (data point value is −0.078) for the 10 cells/ml concentration of MPA samples. TTC, tetrazolium chloride.

We tested how sterilized coral water from the common corals *A. millepora*, *P. cylindrica*, and *P. damicornis* affected the metabolic activity of *V. coralliilyticus* as a function of temperature (24° and 28°C), inoculum concentration of the pathogen, and whether corals came from coral-dominated MPAs or algae-dominated fished areas. Coral water significantly altered *V. coralliilyticus* activity with the effect varying considerably on the basis of coral species, temperature, and the concentration of the *V. coralliilyticus* inoculum (Fig. 1 and table S1, A and B; linear mixed-effects model for each species). *P. cylindrica* inhibited activity by 94 to 98% at 24°C and 35 to 94% at 28°C; *A. millepora* inhibited activity by 61 to 87% at 24°C and 22 to 68% at 28°C; and *P. damicornis* inhibited activity by 8 to 19% at 24°C but stimulated activity by 6 to 12% at 28°C. Suppression of *V. coralliilyticus* declined with increasing inoculum concentration for all corals but especially for *P. cylindrica* at 28°C.

For *A. millepora*, inhibition differed significantly between individuals from coral-dominated MPAs versus macroalgae-dominated fished areas (linear mixed-effects model; Fig. 1, C and D) especially at the higher temperature. At 28°C, coral water of *A. millepora* from MPAs was 75 to 154% more inhibitory than coral water from individuals in fished areas (Fig. 1D and fig. S1B); this effect persisted across pathogen inoculum densities (fig. S1B). At 24°C, patterns were similar but less marked (Fig. 1C and fig. S1A).

**Fig. 2:**
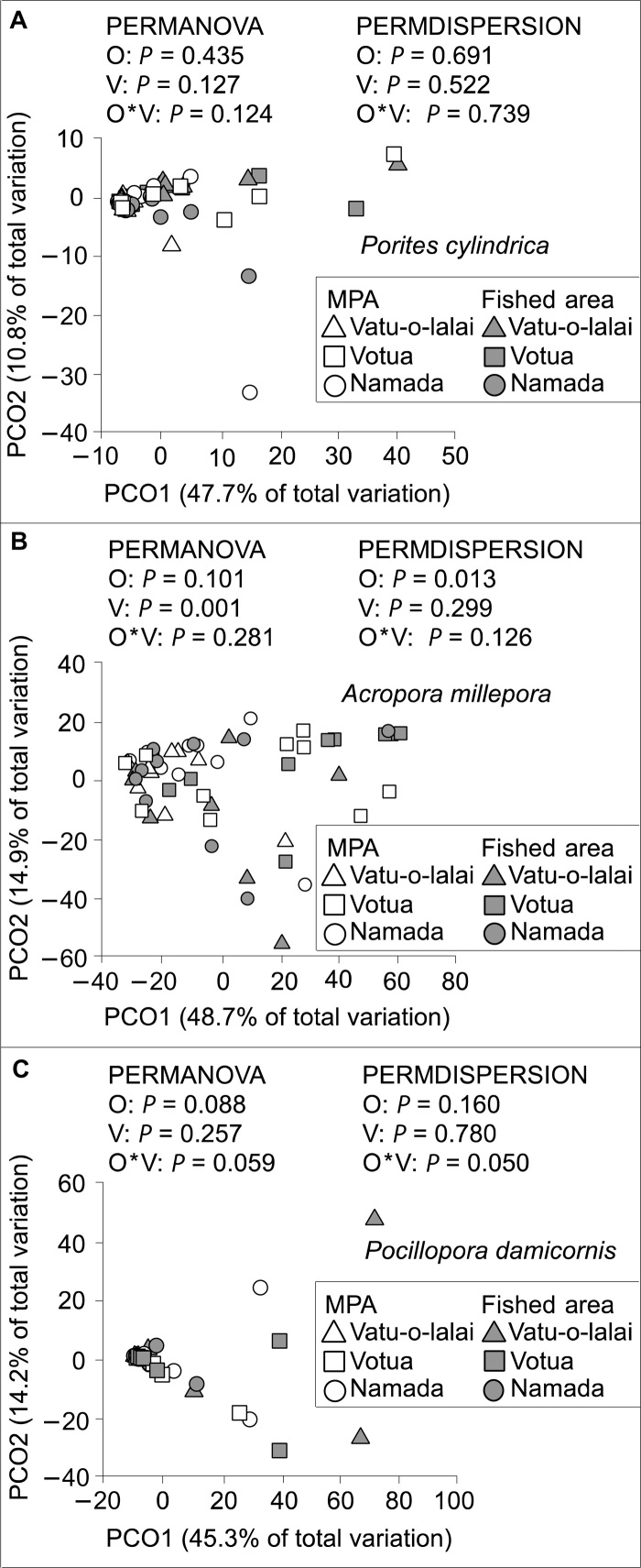
Principal coordinate analysis plots with PERMANOVA and PERMDISPERSION tests of microbial community composition and dispersion on operational taxonomic unit tables rarefied to a uniform sequencing depth of 7700 sequences per sample. (**A**) *P. cylindrica* (*n* = 28, 30 for MPA and fished-area coral). (**B**) *A. millepora* (*n* = 29, 28 for MPA and fished-area coral). (**C**) *P. damicornis* (*n* = 26, 23 for MPA and fished-area coral). Abbreviations O and V represent factors origin (MPA or fished area) and village, respectively. PCO, principal coordinate analysis.

Composition of coral microbiomes differed significantly among coral species [permutational multivariate analysis of variance (PERMANOVA) *P* = 0.001] but not between conspecifics from MPAs and fished areas (Fig. 2 and fig. S2). The large difference in anti-pathogen potency of *A. millepora* from MPAs versus fished areas was not paralleled by a significant difference in the coral’s microbiome between areas. There were no between-habitat type differences in the composition of *A. millepora* microbiomes regardless of whether all *A. millepora* samples were tested together via two-factor analysis (PERMANOVA Origin *P* = 0.101; Fig. 2B) or independently at each village by one-factor analysis (PERMANOVA on factor Origin at Namada, *P* = 0.679; at Vatu-o-lalai, *P* = 0.111; at Votua, *P* = 0.132). Microbiome composition of *A. millepora* differed among villages (PERMANOVA Village *P* = 0.001; Fig. 2B), but collections across villages were taken at different times, making it impossible to distinguish location versus time effects. However, in *A. millepora* (where anti-pathogen activity was weaker in fished areas), dispersion in microbiome composition was significantly higher in fished areas compared to MPAs (PERMDISPERSION Origin *P* = 0.013; Fig. 2B) and also elevated in comparison to the other two coral species (table S2, B and C). For *P. cylindrica* and *P. damicornis*, neither microbiome dispersion (Fig. 2, A and C) nor anti-pathogen activity differed between areas (Fig. 1).

Alpha diversity [operational taxonomic unit (OTU) richness and Shannon diversity] did not differ between coral microbiomes from MPAs and fished areas except for *P. damicornis*, which exhibited higher OTU richness in fished areas (78 to 135 OTUs) compared to MPAs (68 to 90 OTUs; fig. S3E). This difference was not associated with a difference in *P. damicornis* anti-pathogen potency between corals from MPAs versus fished sites. The microbiomes of all three coral species were characterized by >80% relative abundance of OTUs in the Endozoicimonaceae (Gammaproteobacteria, total of 1404 OTUs across datasets; fig. S2 and table S3) with each species harboring a unique Endozoicimonaceae community (PERMANOVA coral species *P* = 0.001 overall and for each pairwise test *P* = 0.001). Indicator analyses identified Endozoicimonaceae OTU 987 as an indicator of *A. millepora* from MPAs (specificity, 85.9%; fidelity, 55.2%). OTU 922761 of the Enterobacteriaceae (Gammaproteobacteria) was an indicator of fished-area *A. millepora* (specificity, 89.7%; fidelity, 50.0%), and OTU 823476 of the genus *Alteromonas* (Gammaproteobacteria) was an indicator of fished-area *P. damicornis* (specificity, 97.7%; fidelity, 82.6). Significant indicator OTUs were not detected for *P. cylindrica*.

Despite detecting differences in *A. millepora* anti-pathogen activity between MPA and fished areas, we did not detect differences in relative abundance, OTU richness, or Shannon diversity of Vibrionaceae associated with *A. millepora* from these areas (tables S4 and S5). Relative abundance of Vibrionaceae associated with *P. cylindrica* also did not differ between MPAs and fished areas (table S4). In *P. damicornis*, however, Vibrionaceae relative abundance was ~18-fold higher in fished areas compared to MPAs (1.66 versus 0.09%; Origin, *P* = 0.032; Village, *P* = 0.129; Origin*Village, *P* = 0.059; table S4), but this coral did not differ in anti-pathogen activity between these areas. We did not detect any *V. coralliilyticus* sequences in our coral samples, regardless of species or area.

Despite the lack of intraspecific differences in community composition of coral microbiomes between coral-dominated versus macroalgae-dominated sites, the taxonomic composition of microbiomes from benthic water (water from within 1 cm of hard bottom surfaces) did differ significantly between MPAs and fished areas, as well as between villages (PERMANOVA: Origin, *P* = 0.001; Village, *P* = 0.001; Origin*Village, *P* = 0.001; fig. S4). We identified 269 OTUs as indicators of MPA benthic water and 502 OTUs as indicators of fished-area water. OTU richness was greater in fished areas (3215 to 3488 OTUs) than MPAs (2875 to 2986 OTUs) (two-factor permutation ANOVA: Origin, *P* = 0.018; fig. S5A). Shannon diversity and among-sample dispersion in microbiome composition of benthic water did not differ on the basis of reef state or village site (permutation two-factor ANOVA; figs. S4 and S5B).

## DISCUSSION

Coral reefs are undergoing precipitous decline, with functionally and evolutionarily critical species, such as acroporids, often exhibiting the greatest losses (*12*, *36*). Previous studies have suggested that coral microbiomes are critical in protecting corals from pathogenic microbes (*5*, *6*). Thus, it is concerning that coral microbiomes may be compromised by elevated temperatures (*5*, *6*, *19*). Macroalgae also may interact with elevated temperatures to chemically destabilize and alter coral microbiomes (*9*, *19*). Here, we show that (i) all three corals we tested had defenses against a coral-bleaching pathogen; (ii) this defense was more effective at 24°C than at 28°C; (iii) establishment of protected areas bolstered anti-pathogen defense of an ecologically important and sensitive coral genus, *Acropora*, but not the resistant genus *Porites* [one of the most persistent genera on degraded reefs (*37*)] or the weedy genus *Pocillopora* [often one of the first to colonize reefs after disturbances (*38*)]; (iv) the compromised potency of coral water from *Acropora* collected in the macroalgae-dominated fished areas compared to the coral-dominated MPAs coincided with different indicator strains (within Enterobacteriaceae versus Endozoicimonaceae) and with increased microbiome variability rather than with large-scale changes in microbiome community composition; and (v) despite benthic waters from MPA and fished areas differing in microbiome composition, conspecific corals in these areas had similar microbiomes, suggesting that corals are regulating their microbiomes despite very dissimilar microbial environmental settings.

All three corals suppressed the pathogen *V. coralliilyticus* at 24°C, but suppression was compromised to varying degrees at 28°C. Because we tested the effects of sterilized coral water rather than effects of the living coral or its living microbiome, the observed suppression suggests the presence of coral-associated chemical defenses against *V. coralliilyticus*. Alternatively, it is possible that some bacteriophages may be able to withstand our sterilization procedures and contribute to the suppression of *V. coralliilyticus*. However, exposure to UV-C irradiation (as we did in our sterilization procedures) is effective at rapidly inactivating phages across broad taxonomic groups (*39*, *40*), but there is some variance in this effect, and we do not know whether phages in our samples were inactivated by this procedure. In addition, if bacteriophages that attack *V. coralliilyticus* degrade at elevated temperatures (e.g., at 28°C, explaining the lower anti-pathogen activity in our experiments at 28°C), then this would disallow bacteriophages from effectively protecting corals at temperatures that promote rapid growth and infection by this pathogen (*31*, *41*). Thus, plausible explanations for the decline in anti-pathogen defense at 28°C are (i) degradation of active chemistry at elevated temperatures, (ii) more rapid growth of *V. coralliilyticus* at elevated temperatures and the higher density of pathogens binding or degrading the active compounds, (iii) production or up-regulation of *V. coralliilyticus* virulence factors involved in binding and inactivation of anti-pathogen molecules at elevated temperatures, and (iv) host microbiomes supporting bacteriophages that attack *V. coralliilyticus* more effectively at 24°C than 28°C.

Although the potency of *P. cylindrica* and *P. damicornis* defenses was unaffected by reef state (protected versus fished areas), coral water of *A. millepora* from algae-dominated fished reefs exhibited 43 to 61% less inhibition compared to conspecifics from coral-dominated MPAs, and this decline was greater at elevated temperature. The decline of an acroporid coral’s defense against a thermally regulated coral pathogen on algae-dominated reefs is worrisome given that many reefs are shifting from coral to macroalgal dominance and that thermal stress events have increased in frequency by fivefold over the past 40 years (*2*). Acroporids are disproportionately important in generating keystone structures on reefs (*34*), are associated with increased diversity of other critical reef species such as fishes (*35*), and have suffered high losses from ocean warming and disease (*2*, *7*, *12*, *36*). If other acroporid corals respond similarly to changes in reef state, then declines in anti-pathogen defense may contribute to, and perpetuate, losses within this structurally and ecologically critical genus. It is equally interesting that the potency of *P. cylindrica* defenses was high and unaffected by reef states that differed markedly in both macroalgal cover and benthic microbiomes; this genus is commonly one of the most persistent on degraded reefs (*37*). Its resilience may be promoted by its potent suppression of a *Vibrio* pathogen (Fig. 1), but whether this anti-pathogen effect is produced by the host or its symbionts is yet to be determined. Previous investigations of defensive compounds among benthic invertebrates have commonly found potent defenses among soft-bodied invertebrates and that these are often produced by microbial associates (*42*). Potent chemical defenses appear uncommon among hard corals (*23*), but numerous investigations have isolated antibiotic-producing microbes from coral microbiomes (*6*, *8*, *9*).

Despite the microbiome of *A. millepora* not differing in community composition between coral- and macroalgae-dominated areas, *A. millepora* within algae-dominated areas exhibited an increase in microbiome compositional variability (dispersion) relative to conspecifics in the MPAs. This is consistent with the increased variability of host microbiomes in response to stressors that have been noted in a variety of species, including corals, mice, chimpanzees, and humans lending support to the importance of the Anna Karenina principle for coral health (*21*). The increased variability of *A. millepora* microbiomes within fished areas may indicate a decline of regulatory mechanisms that constrain the coral microbiome (*6*, *21*). In support of these hypotheses, *A. millepora* from fished areas were relatively depleted in an indicator strain of Endozoicimonaceae, a group hypothesized to play mutualistic roles in coral health and that is prevalent in healthy individuals of other coral genera (*43*). In contrast, fished-area *A. millepora* were enriched in an indicator bacterium of the family Enterobactericeae, a ubiquitous bacterial family with members previously associated with coral disease (*7*, *44*). The functional significance of these nuanced shifts in OTUs between MPA and fished-area coral is unknown but is consistent with recent investigations (*20*, *45*) suggesting that minor alterations in microbiome composition may be associated with large biotic consequences.

Unlike *A. millepora*, *P. cylindrica* and *P. damicornis* did not differ in their anti-pathogen defense or microbiome variability between macroalgae-dominated fished areas and coral-dominated MPAs. This lack of change for *P. cylindrica* may help explain its common persistence on degraded reefs (*37*). Although *P. damicornis* microbiomes did not differ significantly in community composition between fished and protected areas (Fig. 2C), those from fished areas had higher OTU richness compared to conspecifics from MPAs. Elevated microbiome diversity and richness in response to stressors are common (*9*) and could reflect a decline of regulatory mechanisms or the coral’s adjustment to changing conditions via alterations in its protective microbiome. We also detected higher relative abundances of Vibrionaceae associated with *P. damicornis* from fished areas compared to MPAs, which has been documented previously for this species from our sites (*20*). Coral water from *P. damicornis* exhibits weak anti-pathogen activity toward *V. coralliilyticus* at 24°C; this same coral water is stimulatory at 28°C, but this species does not exhibit differences between MPAs and fished areas in anti-pathogen defense. Thus, this species exhibits weak to no anti-pathogen defense against V. *coralliilyticus. P. damicornis* is a rapidly colonizing weedy species (*38*) and may invest more energy in growth and recruitment than in defense against pathogens.

Despite drastic differences in benthic algal abundances (*28*) and benthic seawater microbiome composition (fig. S4), we did not detect community-level differences in coral microbiome composition between MPAs and fished areas for any of the species investigated. This suggests that corals are regulating their microbiomes in spite of considerable biotic differences in their surroundings. Our results appear to contrast with previous work indicating that macroalgae can cause coral microbiomes to shift toward ones enriched in copiotrophic and virulent bacteria (*9*, *17*). However, we did not sample corals at the boundary of coral and algal contact where these shifts may be concentrated (*15*, *17*). We instead sampled haphazardly from corals growing in macroalgae- versus coral-dominated settings. However, our results from these strongly diverging field sites indicate that marked differences in macroalgal abundance and in the surrounding benthic microbial community may have more nuanced effects on coral microbiomes. These differences in the surrounding biotic community may affect microbial indicator taxa and variance in microbiome composition rather than abundance of dominant community members (fig. S2).

Reef state affected corals in species-specific ways. For an acroporid coral from macroalgae-dominated areas, we detected a decline in anti-pathogen defense against a coral-bleaching pathogen, loss of a potentially beneficial symbiont in the Endozoicimonaceae, and increases in microbiome variability. In contrast, anti-pathogen defense and microbiome variability did not differ in *P. damicornis* and *P. cylindrica* between fished areas and MPAs, suggesting greater resistance to declines in reef state for these species. Regardless of collection site, the microbiomes of all three corals were dominated by Endozoicimonaceae with each coral harboring a distinct Endozoicimonaceae community. Host-specific Endozoicimonaceae compositions may therefore play a role in coral resistance to pathogens, either via production of antimicrobials, by providing amino acids to the host (*9*), or altering host microbial community dynamics in other ways. Only nine Endozoicimonaceae occurred at ≥1% average relative abundance, making them likely candidates for tests of their functional roles in coral microbiomes. OTU 987 was an indicator of *A. millepora* from MPAs; this association is correlational only but suggests that this indicator bacterium could be worth evaluating to determine whether it plays a role in the elevated anti-pathogen activity of *A. millepora* from MPAs.

It is presently uncertain whether the antibiotic effects documented for all three corals, or the variable effects documented for *A. millepora* from MPAs versus fished areas, are due to host- or symbiont-produced chemicals. We also cannot exclude the possibility that antibiotic effects might result from bacteriophages that were present on corals but resistant to our sterilization procedures. However, the effectiveness of UV-C irradiation at suppressing most viruses (*39*, *40*) and the decline in inhibitory activity with warmer temperatures, near the temperature optimum for *V. coralliilyticus*, suggest that mechanisms other than bacteriophages may be a more plausible explanation for alterations of anti-pathogen activity. If anti-pathogen activity is due to chemical defenses, our assays are likely conservative in terms of documenting potency. Our collection method involved dilution of any coral-associated metabolites with 50 ml of seawater. In addition, live organisms often produce chemical defenses continuously. Our assays were conducted using a single dose; this dosage likely declined as it was metabolized or degraded over the course of storage and deployment in our assays. Thus, the anti-pathogen effects observed in vitro could be diminished compared to those occurring on living corals. However, it is also possible that the process of fragmenting and agitating corals may induce production or release of antibacterial compounds (*46*) from the coral host or its microbial symbionts. Geffen and Rosenberg (*46*) found that 10 min of mechanical stress induced production of antimicrobials by *P. damicornis*; we agitated our corals for only 20 s. Activity from the Geffen and Rosenberg method was found to be much more potent than the activity in our experiments. In their assays, for example, 99% of *V. coralliilyticus* were killed within only 6 min of exposure to water from mechanically stressed coral (*46*). In contrast, we document more modest activity for *P. damicornis* after 24 hours of incubation with coral water collected using our methodology. Determining the compounds (or bacteriophages) responsible for coral anti-pathogen effects, their effective concentration (or density), their origin (host or microbially produced), and their potency in situ and across other host taxa would be useful.

## CONCLUSION

Processes such as ocean warming, overfishing, pollution, and other anthropogenic stresses not only suppress corals and advantage macroalgae but also create positive feedbacks that suppress coral reef resilience (*3*, *4*). Acroporid corals are disproportionately important for reef recovery. They provide the critical topographic complexity (*34*) that facilitates ecological retention and evolutionary diversification of numerous groups of reef fishes (*34*, *35*) and rapid growth make acroporids critically important for maintaining reef accretion (*37*). These corals are also among the most threatened, and rapidly declining, due to bleaching and disease (*7*, *36*). Here, *A. millepora* exhibited a decline in defense against *V. coralliilyticus* and increased microbiome variability, consistent with the Anna Karenina principle, when collected from macroalgae-dominated fished reefs versus coral-dominated MPAs protected from fishing. In contrast, anti-pathogen defense and microbiome variability did not differ for the hardy species, *P. cylindrica*, or the weedy species, *P. damicornis*. *P. cylindrica* strongly suppressed *V. coralliilyticus*, and this coral, along with others in the genus, is among the most common corals on degraded reefs and is resilient to stressors that often harm other coral taxa. However, reefs composed of only a few resistant corals, such as *Porites*, will provide neither the keystone structures needed by other reef species (*34*) nor the functional advantages generated by coral biodiversity (*47*). At our study sites, recovery of herbivores and declines in macroalgae following protection from fishing (*27*, *28*) were associated with 75 to 154% greater inhibition of *V. coralliilyticus* at 28°C by *A. millepora*. This suggests that local management to improve reef state (e.g., protecting fishes that reduce macroalgal cover) may also bolster anti-pathogen defenses against climate-induced coral pathogens for critical acroporid species during warming events.

## METHODS

### Sites and species

We investigated how reef state affected coral microbiomes and defense against the coral pathogen *V. coralliilyticus* [ATCC (American Type Culture Collection) BAA-450] using the common corals *P. cylindrica*, *A. millepora*, and *P. damicornis*. Corals were collected haphazardly throughout three colocated pairs of small (0.5 to 0.8 km^2^), no-take MPAs and their adjacent fished reefs at Namada (18°11.30′S, 177°37.10′E), Vatu-o-lalai (18°12.26′S, 177°41.26′E), and Votua (18°13.08′S, 177°42.59′E) villages along the southwest coast of Viti Levu, Fiji (fig. S6) between October and December of 2014 (8 to 12 December, 21 to 24 October, 25 to 29 October, respectively at each village). We did not measure water temperature while collecting samples, but shallow water temperatures in Fiji during this period generally range from 25° to 28°C with considerable variance among and within days due to tidal height and local weather patterns affecting these back-reef lagoons. Protected areas were established in 2002 (Vatu-o-lalai, Namada) and 2003 (Votua). Protection within the MPAs in the decade before our study resulted in drastic differences in benthic communities between MPAs and fished areas at each village (*27*, *28*, *30*). Mass of herbivorous fishes was 7 to 17× greater in MPAs versus fished areas and MPAs had 38 to 56% coral cover and ≤3% macroalgal cover, while fished areas only had 4 to 16% coral cover but 50 to 90% macroalgal cover on hard substrates (*28*).

We collected two to three individuals for each coral species from MPAs and adjacent fished areas each day to assure that samples across species and between MPA and fished areas were interspersed in time. We repeated this over multiple days until we acquired a total of 10 individuals for species from each area at each village. This allowed us to test for differences in coral traits between MPAs and fished areas, with these areas replicated across three pairs of MPA and fished areas. We could not collect from all villages at the same time; thus, village and time are confounded and should be interpreted with caution.

### Anti-pathogen activity of corals

Many marine invertebrates harbor microbes that produce host-associated chemical defenses (*42*). We therefore investigated anti-pathogen activity in corals and evaluated how this related to changes in the coral’s microbiome. Most studies of marine chemical defenses conduct exhaustive extraction of host tissues using multiple rounds of extraction with strong solvents that can last upward of 24 hours for each extraction (*48*–*50*). After removal of the solvents, these extracts are then tested against pathogens or predators. In contrast to these exhaustive methods, we simply agitated coral samples in unfiltered reef water for 20 s. We used a hammer and chisel to collect 50-ml displacement volume of coral per colony (*n* = 10 colonies per species from within each of the three MPAs and three fished sites). Each coral sample was volumetrically displaced in a 1:1 ratio with reef water collected from within 0.5 m of each colony and agitated in a 250-ml wide-mouth glass jar (VWR Radnor, PA) for 20 s. Glass jars were washed with 70% ethanol and deionized water (3×) between samples. Samples generated by this method are referred to as coral water. Coral water contains reef water, coral mucus, and anything else released during coral agitation, such as antibacterial compounds (*46*). Samples were decanted into a 50-ml sterile polystyrene tube and frozen at −20°C until used in bioassays with *V. coralliilyticus* (BAA-450).

Because the seawater that we used from each site to generate the coral water sample might itself have contained anti-pathogen activity, we also collected 50 ml of reef water adjacent to each colony (frozen as above) as a control for our bioassays with *V. coralliilyticus*. A random subset of three coral water samples and their corresponding control (reef water) samples from each of the three MPAs and each of the three fished areas (i.e., *n* = 9 samples per species per area type) were selected for bioassays against *V. coralliilyticus*.

For the bioassay, 100 μl of coral water, or adjacent reef water (the control), was aliquoted per well in sterile 96-well round-bottom plates, lyophilized on a freeze dryer, and UV-C–irradiated (TUV30W G30T8, Philips Amsterdam, The Netherlands) for 90 s to kill any living microbial cells that survived lyophilization. While viral load also declines rapidly with UV irradiation (*39*, *40*), it is possible that some bacteriophages or other viruses could survive our pretreatment and contribute to biological effects observed in our experiments. Regardless of the source of an antibiotic effect, this method should produce a conservative estimate of the holobiont’s defense against *V. coralliilyticus* because it introduces a single, diluted dose (if the defense is chemical) that may degrade or be metabolized during the assay. If activity is due to bacteriophages, viral load should have been considerably reduced, or possibly eliminated, by UV-C irradiation (*39*, *40*). In nature, the holobiont would presumably be continuously producing chemical defenses or harboring more dense and more viable populations of phages than in our experiments, which implemented freeze drying and UV-C irradiation. To the samples of dried coral water in the bottom of each well, we then added 100 μl of *V*. *coralliilyticus* bacterial cell suspension. These inoculum cultures were grown in marine broth (Difco 2216, Becton Dickinson, Franklin Lakes, NJ) and added to the wells during log growth to span a gradient of cell densities in 96-well plates from 10 to 1000 cells/ml (with additional concentrations of 10,000 to 1,000,000 cells/ml for *A. millepora* only, after detecting differences with reef state at lower bacterial concentrations).

Laboratory assays were conducted at temperatures of 24° and 28°C within incubators without shaking. These temperatures are near the upper and lower limits of the seasonal means in Fiji and represent temperatures at which *V. coralliilyticus* is less (24°C) versus more (28°C) virulent (*10*). 2,3,5-Triphenyl tetrazolium chloride (TTC; TCI America Portland, Oregon) was added (0.05 μg/μl final concentration) to each well, and the plates were incubated for 24 hours. Reduction of TTC by cellular respiration produces a red compound, triphenylformazan, allowing a direct measurement of metabolic activity. We used this method rather than measuring turbidity associated with cell density or direct cell counts because density assesses both live and dead, healthy and unhealthy cells, whereas this method assesses only those cells that are metabolically active. Reduction of TTC by *V*. *coralliilyticus* was quantified by absorbance at 490 nm using a BioTek ELx800 absorbance reader (BioTek, Winooski, VT). Background absorbance was measured in blanks containing lyophilized and UV-C–irradiated coral water or reef water reconstituted in marine broth with TTC but without bacteria. Blank-corrected measurements were used to determine relative *V*. *coralliilyticus* metabolism, expressed as a ratio of metabolism in the coral water compared to the control (reef water collected adjacent to the coral). Values >1 suggest that coral water is stimulatory, and values <1 suggest inhibition. To statistically evaluate inhibition or stimulation, we compared coral water to reef water with functions aov or aovp in package lm v 2.1.0 implemented within RStudio 3.0 with false discovery rate corrections for multiple comparisons (table S1, A and B). Linear mixed-effects modeling within RStudio 3.0 package nlme v 3.1-137 was implemented to determine whether area of origin (MPA or fished area), temperature (24° or 28°C), and bacterial inoculum concentration (cells per milliliter) influenced each species anti-pathogen activity (via relative metabolism of TTC by *V*. *coralliilyticus*). Akaike information criterion was implemented for model selection.

### DNA extractions and sequencing of the 16*S* ribosomal RNA gene

Fragments of coral (~1 g) from the same colonies used to obtain coral water were preserved in 1 ml of RNAlater (Qiagen, Carlsbad, CA) in the field and stored at −20°C until DNA extraction. Our previous analyses at these sites demonstrated that water collected from ~1 m above the benthos did not differ in microbiome community composition, (*20*) but previous studies at other reefs found microbiome differences in water collected from within ~25 cm of the reef surface on macroalgae- versus coral-dominated reefs (*18*). Thus, we collected benthic water samples from each habitat type to evaluate whether our study reefs differed in some fundamental way from those observed in previous studies. Benthic water samples (*n* = 10 per site) from within 1 cm of the benthos were collected haphazardly at each site using a 240-ml syringe. Water was filtered through a 0.22-μm polyethersulfone filter (total volumes ranged from 100 to 240 ml depending on filter clogging; Millipore, Sigma-Aldrich, St. Louis, MO), which was then preserved in RNAlater and frozen at −20°C.

We performed Illumina sequencing of the 16*S* ribosomal RNA (rRNA) gene to characterize the microbial community in our samples. DNA was extracted from approximately 250 mg of coral using the MoBio PowerSoil Kit and from water filters using the MoBio PowerWater Kit (Qiagen). For each sample, residual RNAlater solution was centrifuged at 9391*g* for 10 min to pellet dissociated microbial cells. This pellet was resuspended with C1 solution and added to a powerbead tube (MoBio Laboratories, Qiagen). Dual-barcoded primers (F515 and R806) appended with Illumina sequencing adapters [see Kozich *et al.* (*51*)] were used to amplify the V4 region of the microbial 16*S* rRNA gene. Polymerase chain reactions (PCRs) were carried out in triplicate. Total reaction volume was 50 μl, containing 45 μl of Platinum PCR SuperMix (Life Technologies, Thermo Fisher Scientific, Waltham, MA), 1 μl each of forward and reverse primers (final concentration, 0.2 μm), and 3 μl of template DNA. Thermal cycling involved initial denaturation at 94°C (3 min), 35 cycles of denaturation at 94°C (45 s), primer annealing at 50°C (45 s), primer extension at 72°C (90 s), and final extension at 72°C (10 min). SequalPrep plates (Thermo Fisher Scientific, Waltham, MA) were used to remove impurities and normalize DNA concentrations across samples. Pooled amplicons were sequenced on an Illumina MiSeq (Illumina Inc., San Diego, CA) in-house using a 500-cycle kit (250 × 250 nucleotides) spiked with 10% PhiX to introduce sequence diversity.

### Data availability

Raw sequence reads were deposited at National Center for Biotechnology Information (NCBI; bioproject number PRJNA476581).

### Microbiome data analyses

TrimGalore! (www.bioinformatics.babraham.ac.uk/projects/trim:galore/) was used to demultiplex and trim [100–base pair (bp) cutoff length] sequence reads and to remove low-quality reads (Phred score cutoff, 25). FLASH (*52*) was used to merge paired-end reads using the criteria: read length, >250 bp; fragment length, >300 bp; fragment SD, <30 bp. Chimeras were identified and removed with USEARCH (*53*) in QIIME (*54*). Remaining sequences were then clustered into OTUs with 97% similarity clusters using the UCLUST algorithm (*53*), followed by open-reference picking to assign taxonomy based on the Greengenes database 13.8 (*55*, *56*). After quality filtering, 12,057,258 sequences remained from 21,520,596 sequences generated by the MiSeq run with per-sample counts ranging from 44 to 131,802 for benthic water samples and 1191 to 216,258 for coral samples. Microbiome analyses were performed on OTU tables after rarefaction to a uniform sequence count of 7700 for coral samples and 17,700 for water samples (fig. S7).

Alpha (number of OTUs, Shannon diversity) and beta diversity (Bray-Curtis dissimilarity) were calculated in QIIME (*54*). Aov or aovp functions in package lm v 2.1.0 implemented within RStudio 3.0 were used to test for differences in alpha diversity between coral or water samples from MPAs or fished areas by a two-factor ANOVA or permutation ANOVA if data were heteroscedastic (factor 1, Origin; factor 2, Village, where Village is confounded in time). Upon detecting differences in microbiome composition in *A. millepora* among villages, we independently tested for area-of-origin effects via a one-factor PERMANOVA test at each village. Principal coordinate analysis and corresponding tests for differences in microbiome composition (PERMANOVA) and variability (PERMDISPERSION) were implemented in Primer E (*57*) for coral and water samples via two-factor tests (factor 1, Origin; factor 2, Village). Upon detecting differences in microbiome composition in *A. millepora* among villages, we independently tested for area-of-origin effects via a one-factor PERMANOVA test at each village. Coral OTU tables were also filtered to obtain only Endozoicimonaceae OTUs (via QIIME script filter_otus.py) and tested for differences in Endozoicimonaceae OTU communities for each coral species via two-factor (area of origin and village) PERMANOVA and PERMDISPERSION in Primer E. Multilevel pattern analysis was implemented within the indicspecies package v 1.7.6 implemented within RStudio 3.0 to test for MPA or fished-area indicator OTUs for coral species and water samples. OTUs were considered as indicators if their fidelity value was 0.50 or greater. *A. millepora* OTU tables were also filtered to obtain only Vibrionaceae OTUs (via QIIME script filter_otus.py). Aov or aovp functions within RStudio 3.0 package lm v 2.1.0 were used to test for differences in Vibrionaceae alpha diversity (i.e., OTU richness and Shannon diversity, for *A. millepora* only, where we detected differences in anti-pathogen activity) by two-factor ANOVA or permutation ANOVA if data were heteroscedastic (factor 1, Origin; factor 2, Village). Functions aov or aovp within RStudio 3.0 package lm v 2.1.0 were also implemented to test for differences in relative abundances of Vibrionaceae for all three coral species with a two-factor ANOVA or permutation ANOVA if data were heteroscedastic (factor 1, Origin; factor 2, Village).

## Supplementary Material

http://advances.sciencemag.org/cgi/content/full/5/10/eaay1048/DC1

Download PDF

Variable effects of local management on coral defenses against a thermally regulated bleaching pathogen

## SUPPLEMENTARY MATERIALS

Supplementary material for this article is available at http://advances.sciencemag.org/cgi/content/full/5/10/eaay1048/DC1 Fig. S1. Anti-pathogen activity of coral water from *Acropora millepora*. Fig. S2. Average microbial community composition from data rarefied to 7700 sequences per sample for *A. millepora* (*n* = 29, 28 MPA and fished-area coral), *P. damicornis* (*n* = 26, 23 MPA and fished-area coral), and *P. cylindrica* (*n* = 28, 30 for MPA and fished-area coral). Fig. S3. Alpha diversity of corals from MPAs and fished areas. Fig. S4. Principal coordinate analysis with PERMANOVA and PERMDISPERSION tests of microbial community composition and dispersion for benthic water samples on OTU tables rarefied to a uniform sequencing depth of 17,700 sequences per sample (*n* = 27, 18 for MPA and fished-area samples). Fig. S5. OTU richness and diversity of benthic water from each reef site. Fig. S6. Map of MPAs (in red) and fished areas (in blue) used in collection of coral and water samples along the coral coast of Viti Levu, Fiji. Fig. S7. OTU rarefaction curves for each coral and for benthic water from each reef site. Table S1. Statistical contrast values for data shown in Fig. 1 and fig. S1. Table S2. PERMANOVA and PERMDISPERSION results for coral microbial community composition and dispersion. Table S3. Coral microbial community composition. Table S4. Relative abundance and analyses of Vibrionaceae for each coral species and site. Table S5. Diversity of Vibrionaceae.
