# Supplementary material for: Variable effects of local management on coral defenses against a thermally regulated bleaching pathogen
Source: Sci Adv. 2019 Oct 2;5(10):eaay1048. doi: 10.1126/sciadv.aay1048 (PMC6774716; doi:10.1126/sciadv.aay1048)
Supplement: http://advances.sciencemag.org/cgi/content/full/5/10/eaay1048/DC1 [file supp_5_10_eaay1048__index.html]

Science Advances | Science AdvancesAAASSearchScience AdvancesMenu

## Supplementary Materials

**This PDF file includes:**

- Fig. S1. Anti-pathogen activity of coral water from *Acropora millepora*.
- Fig. S2. Average microbial community composition from data rarefied to 7700 sequences per sample for *A. millepora* (*n* = 29, 28 MPA and fished-area coral), *P. damicornis* (*n* = 26, 23 MPA and fished-area coral), and *P. cylindrica* (*n* = 28, 30 for MPA and fished-area coral).
- Fig. S3. Alpha diversity of corals from MPAs and fished areas.
- Fig. S4. Principal coordinate analysis with PERMANOVA and PERMDISPERSION tests of microbial community composition and dispersion for benthic water samples on OTU tables rarefied to a uniform sequencing depth of 17,700 sequences per sample (*n* = 27, 18 for MPA and fished-area samples).
- Fig. S5. OTU richness and diversity of benthic water from each reef site.
- Fig. S6. Map of MPAs (in red) and fished areas (in blue) used in collection of coral and water samples along the coral coast of Viti Levu, Fiji.
- Fig. S7. OTU rarefaction curves for each coral and for benthic water from each reef site.
- Table S1. Statistical contrast values for data shown in Fig. 1 and fig. S1.
- Table S2. PERMANOVA and PERMDISPERSION results for coral microbial community composition and dispersion.
- Table S3. Coral microbial community composition.
- Table S4. Relative abundance and analyses of Vibrionaceae for each coral species and site.
- Table S5. Diversity of Vibrionaceae.

Download PDF

**Files in this Data Supplement:**

- Adobe PDF - aay1048\_SM.pdf
