# Supplementary material for: Variable effects of local management on coral defenses against a thermally regulated bleaching pathogen
Source: Sci Adv. 2019 Oct 2;5(10):eaay1048. doi: 10.1126/sciadv.aay1048 (PMC6774716; doi:10.1126/sciadv.aay1048)
Supplement: Download PDF [file aay1048_SM.pdf]

Science Advances
AAAS

advances.sciencemag.org/cgi/content/full/5/10/eaay1048/DC1

Supplementary Materials for

Variable effects of local management on coral defenses against a thermally regulated bleaching pathogen

Deanna S. Beatty, Jinu Mathew Valayil, Cody S. Clements, Kim B. Ritchie, Frank J. Stewart, Mark E. Hay*

*Corresponding author. Email: mark.hay@biology.gatech.edu

Published 2 October 2019, *Sci. Adv.* **5**, eaay1048 (2019)
DOI: 10.1126/sciadv.aay1048

This PDF file includes:

Fig. S1. Anti-pathogen activity of coral water from *Acropora millepora.*
Fig. S2. Average microbial community composition from data rarefied to 7700 sequences per sample for *A. millepora* (*n* = 29, 28 MPA and fished-area coral), *P. damicornis* (*n* = 26, 23 MPA and fished-area coral), and *P. cylindrica* (*n* = 28, 30 for MPA and fished-area coral).
Fig. S3. Alpha diversity of corals from MPAs and fished areas.
Fig. S4. Principal coordinate analysis with PERMANOVA and PERMDISPERSION tests of microbial community composition and dispersion for benthic water samples on OTU tables rarefied to a uniform sequencing depth of 17,700 sequences per sample (*n* = 27, 18 for MPA and fished-area samples).
Fig. S5. OTU richness and diversity of benthic water from each reef site.
Fig. S6. Map of MPAs (in red) and fished areas (in blue) used in collection of coral and water samples along the coral coast of Viti Levu, Fiji.
Fig. S7. OTU rarefaction curves for each coral and for benthic water from each reef site.
Table S1. Statistical contrast values for data shown in Fig. 1 and fig. S1.
Table S2. PERMANOVA and PERMDISPERSION results for coral microbial community composition and dispersion.
Table S3. Coral microbial community composition.
Table S4. Relative abundance and analyses of Vibrionaceae for each coral species and site.
Table S5. Diversity of Vibrionaceae.

Supplemental Figures:

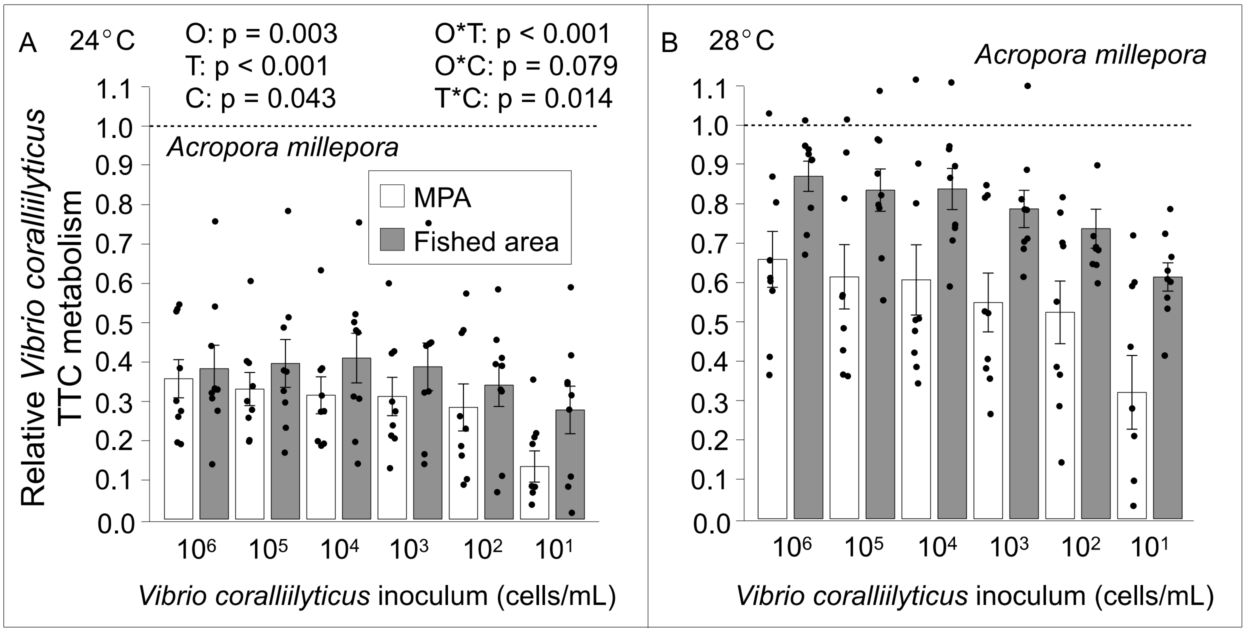

Fig. S1. Anti-pathogen activity of coral water from *Acropora millepora*. Mean (±SE) activity of coral water relative to reef water against *V. coralliilyticus* (quantified by metabolism of tetrazolium chloride [TTC]) at 24°C (**A**) and 28°C (**B**) for *Acropora millepora* as a function of pathogen inoculum concentration. The dashed line at 1.0 is the expected value if there is no effect. *P*-values are from a linear mixed effects model implemented with Akaike Information Criterion (AIC) for model selection (n = 9). Factors remaining after the model selection process are provided. O, T, and C represent origin (MPAs or fished areas), temperature, and concentration of pathogen inoculum, respectively. Dots indicate individual data points. One data point with negative values (after reduction of the optical density of sterilized coral water, see methods for information on data reduction) is not depicted in each of A (data point value is -0.040) and in B (data point value is -0.078) for the 10 cells per mL concentration of MPA samples.

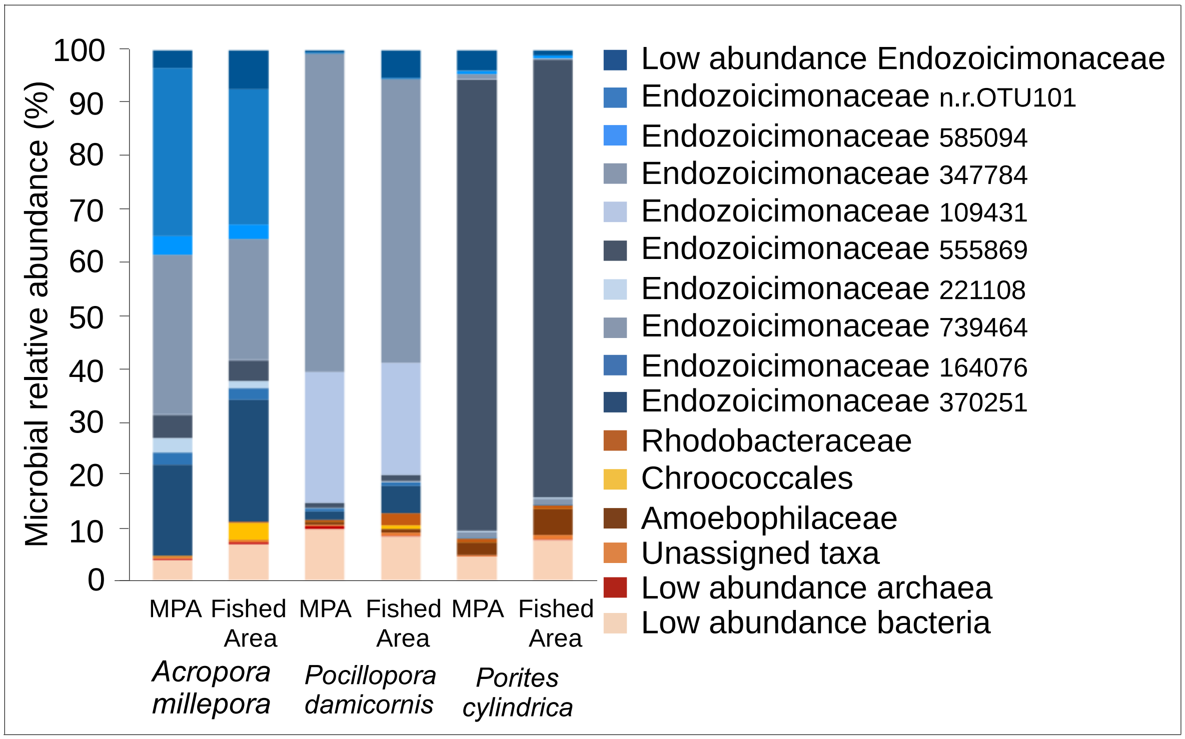

Fig. S2. Average microbial community composition from data rarefied to 7700 sequences per sample for *A. millepora* (*n* = 29, 28 MPA and fished-area coral), *P. damicornis* (*n* = 26, 23 MPA and fished-area coral), and *P. cylindrica* (*n* = 28, 30 for MPA and fished-area coral). Taxa of <2% relative abundance were pooled by domain and depicted as 'low abundance bacteria' and 'low abundance archaea'. Endozoicimonaceae are depicted at the OTU level for taxa contributing to 1% or greater composition, with all remaining Endozoicimonaceae OTUs pooled to generate 'Low abundance Endozoicimonaceae'.

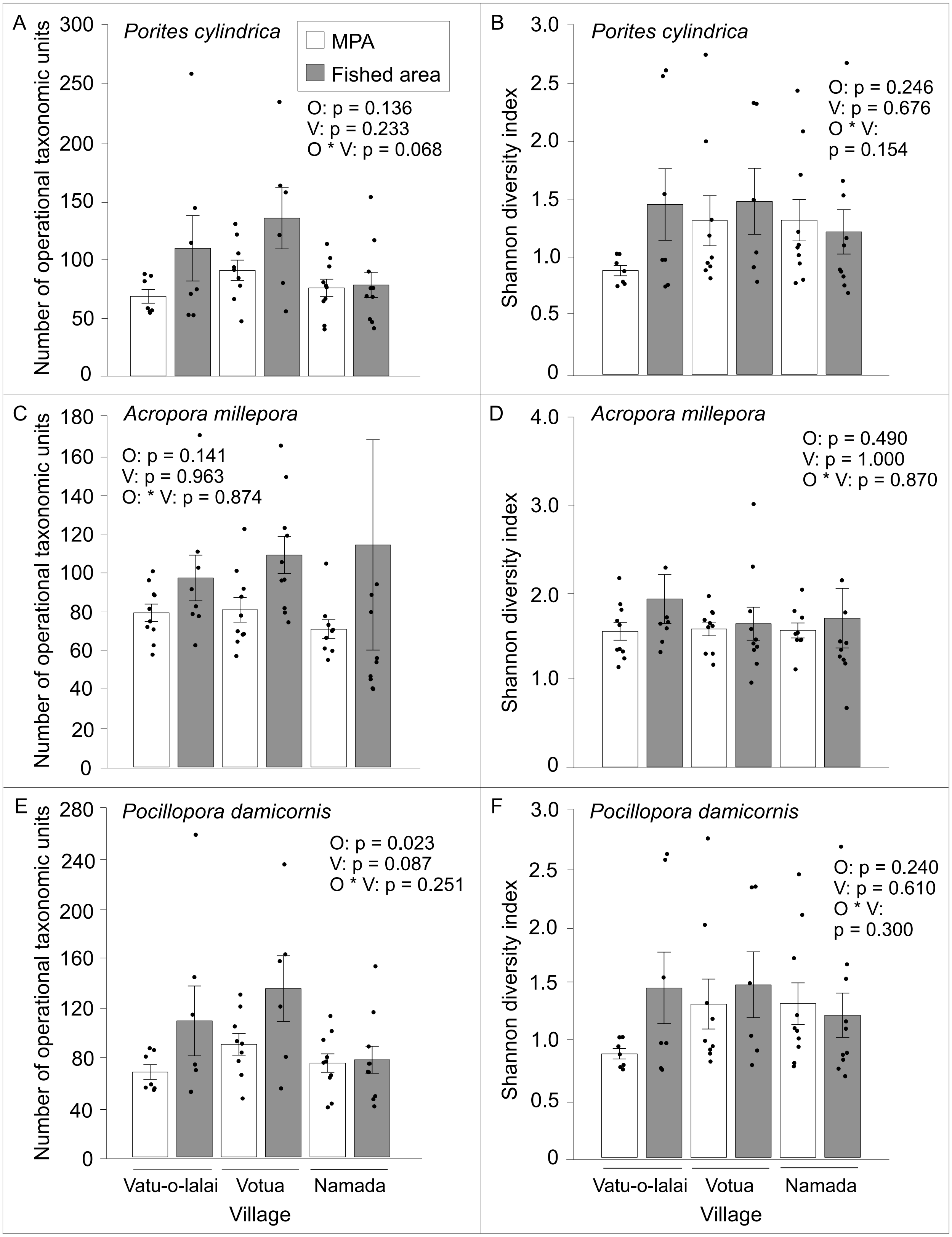

A
Porites cylindrica
MPA
Fished area
O: p = 0.136
V: p = 0.233
O * V: p = 0.068
Number of operational taxonomic units
B
Porites cylindrica
O: p = 0.246
V: p = 0.676
O * V:
p = 0.154
Shannon diversity index
C
Acropora millepora
O: p = 0.141
V: p = 0.963
O: * V: p = 0.874
Number of operational taxonomic units
D
Acropora millepora
O: p = 0.490
V: p = 1.000
O * V: p = 0.870
Shannon diversity index
E
Pocillopora damicornis
O: p = 0.023
V: p = 0.087
O * V: p = 0.251
Number of operational taxonomic units
F
Pocillopora damicornis
O: p = 0.240
V: p = 0.610
O * V:
p = 0.300
Shannon diversity index
Vatu-o-lalai
Votua
Namada
Village

Fig. S3. Alpha diversity of corals from MPAs and fished areas. Graphs on the left show operational taxonomic unit (OTU) richness and on the right show Shannon diversity (mean ± SE) for (**A-B**) *Porites cylindrica* (n = 28, 30 for MPA and fished area coral), (**C-D**) *A. millepora* (n = 29, 28 for MPA and fished area coral), (**E-F**) *Pocillopora damicornis* (n = 26, 23 for MPA and fished area coral). Analyses by two-factor ANOVA or permutation ANOVA. O indicates origin (MPA or fished area) and V represents village. Dots show the individual data points. One (off the graph) data point is not depicted in C and D above for Namada fished area (600 OTUs and 4.583 Shannon diversity).

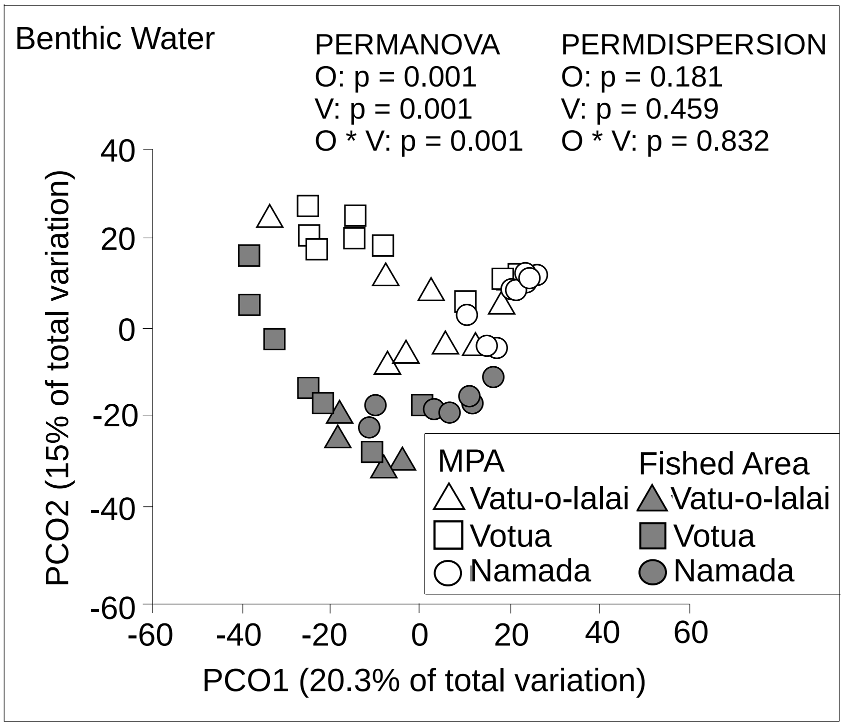

Fig. S4. Principal coordinate analysis with PERMANOVA and PERMDISPERSION tests of microbial community composition and dispersion for benthic water samples on OTU tables rarefied to a uniform sequencing depth of 17,700 sequences per sample (*n* = 27, 18 for MPA and fished-area samples). O and V represent origin and village, respectively, with village confounded by time (i.e., samples in different villages were taken on different dates).

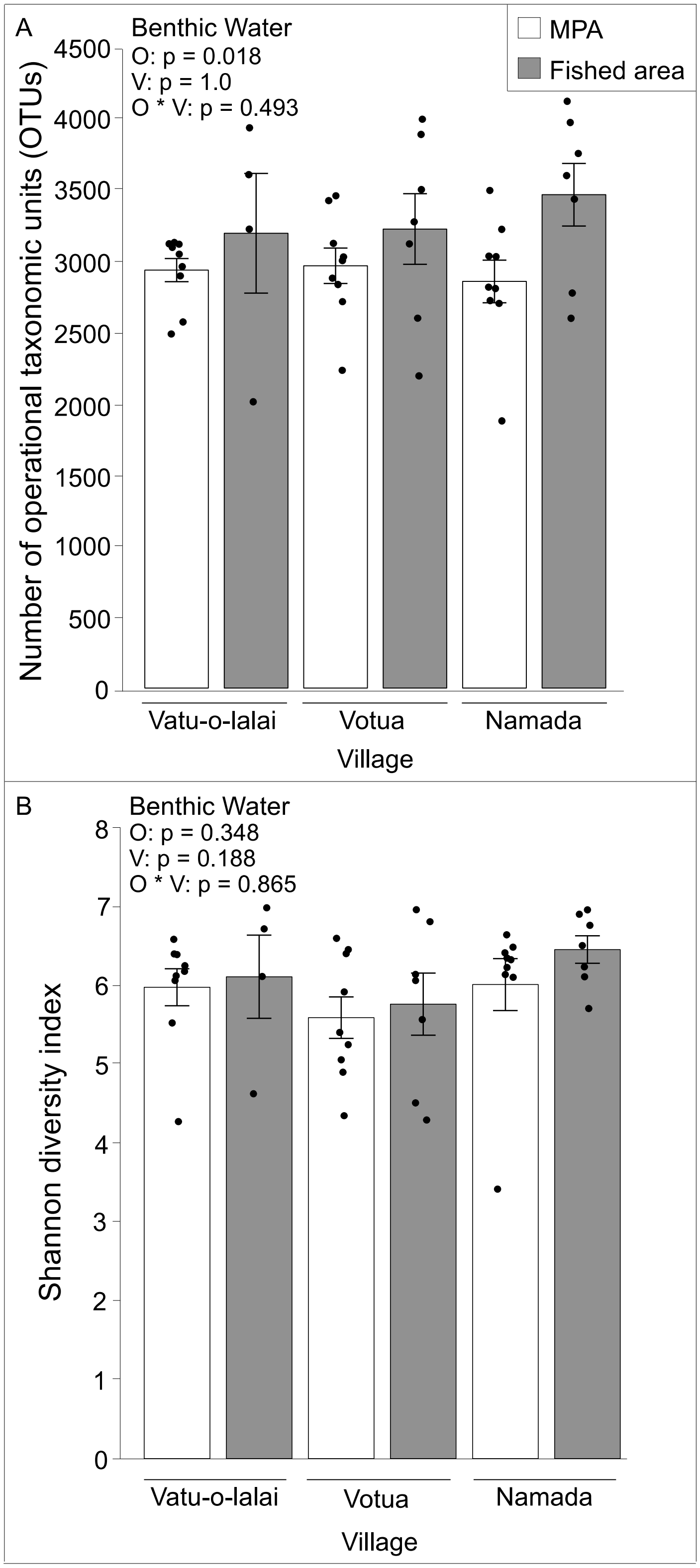

Fig. S5. OTU richness and diversity of benthic water from each reef site. OTU richness (**A**) and Shannon diversity (**B**) for benthic water samples (n = 27, 18 for MPA and fished area samples, respectively). Analyses performed with two-factor permutation ANOVA.

Abbreviations O and V represent factors origin and village, respectively, with village confounded by time.

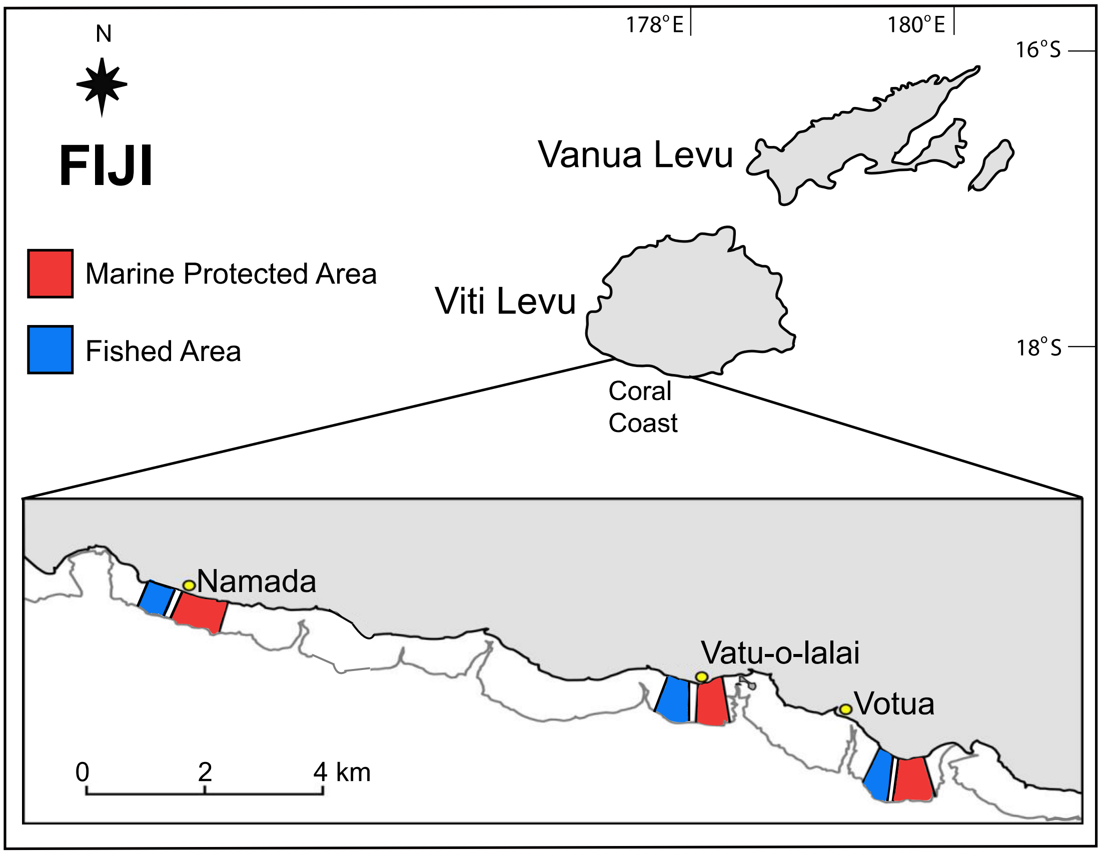

Fig. S6. Map of MPAs (in red) and fished areas (in blue) used in collection of coral and water samples along the coral coast of Viti Levu, Fiji. Image adapted from Figure 1 in (*35*).

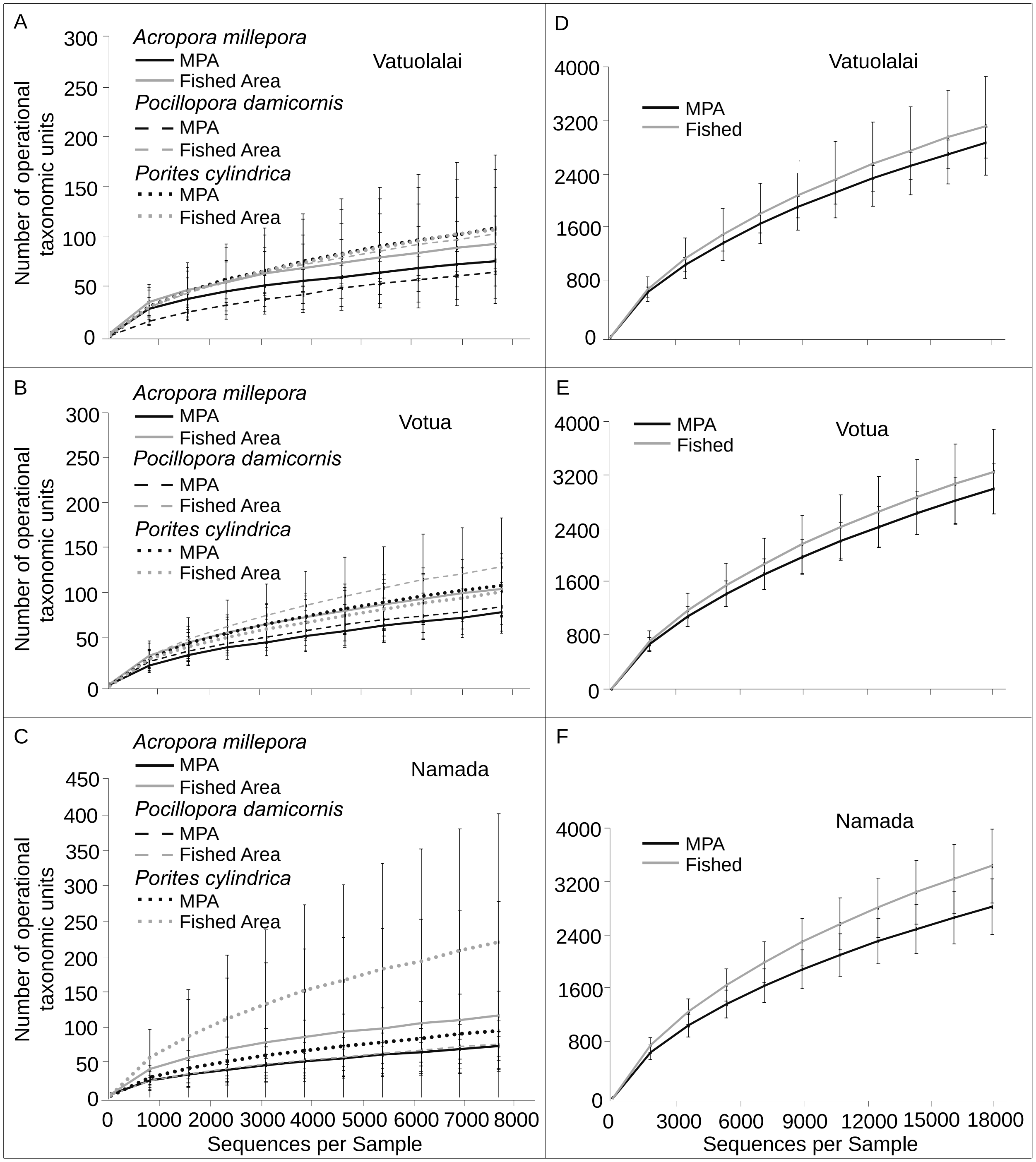

Fig. S7. OTU rarefaction curves for each coral and for benthic water from each reef site.

Rarefaction curves (mean ± SEM) for *Porites cylindrica* (n = 28, 30 for MPA and fished area coral), *A. millepora* (n = 29, 28 MPA and fished area coral), *Pocillopora damicornis* (n = 26, 23

MPA and fished area coral) at each village (**A-C**) and for benthic water (n = 27, 18 MPA and fished area samples) from each village (**D-F**).

Table S1. Statistical contrast values for data shown in Fig. 1 and fig. S1.

Table S1A: Comparisons of treatment (coral water) to control (reef water) at 24C

Coral Species: *Acropora millepora*
Area: MPA
Temperature: 24 Celsius

Concentration of *Vibrio coralliilyticus* cells/mL	ANOVA or permutation ANOVA FDR-corrected p value (coral water compared to seawater)
1000000	<0.001
100000	<0.001
10000	<0.001
1000	<0.001
100	<0.001
10	**<0.001**

Coral Species: *Pocillopora damicornis*
Area: MPA
Temperature: 24 Celsius

Concentration of *Vibrio coralliilyticus* cells/mL	ANOVA or permutation ANOVA FDR-corrected p value (coral water compared to seawater)
1000	**0.192**
100	<0.001
10	0.001

Coral Species: *Porites cylindrica*
Area: MPA
Temperature: 24 Celsius

Concentration of *Vibrio coralliilyticus* cells/mL	ANOVA or permutation ANOVA FDR-corrected p value (coral water compared to seawater)
1000	<0.001
100	<0.001
10	**<0.001**

Coral Species: *Acropora millepora*
Area: Fished Area
Temperature: 24 Celsius

Concentration of *Vibrio coralliilyticus* cells/mL	ANOVA or permutation ANOVA FDR-corrected p value (coral water compared to seawater)
1000000	<0.001
100000	<0.001
10000	**<0.001**
1000	<0.001
100	<0.001
10	<0.001

Coral Species: *Pocillopora damicornis*
Area: Fished Area
Temperature: 24 Celsius

Concentration of *Vibrio coralliilyticus* cells/mL	ANOVA or permutation ANOVA FDR-corrected p value (coral water compared to seawater)
1000	0.092
100	0.038
10	**0.192**

Coral Species: *Porites cylindrica*
Area: Fished Area
Temperature: 24 Celsius

Concentration of *Vibrio coralliilyticus* cells/mL	ANOVA or permutation ANOVA FDR-corrected p value (coral water compared to seawater)
1000	<0.001
100	<0.001
10	**<0.001**

permutation p values are in bold

Table S1B: Comparisons of treatment (coral water) to control (reef water) at 28C

Coral Species: *Acropora millepora*
Area: MPA
Temperature: 28 Celsius

Concentration of *Vibrio coralliilyticus* cells/mL	ANOVA or permutation ANOVA FDR-corrected p value (coral water compared to seawater)
1000000	<0.001
100000	<0.001
10000	<0.001
1000	<0.001
100	<0.001
10	<0.001

Coral Species: *Pocillopora damicornis*
Area: MPA
Temperature: 28 Celsius

Concentration of *Vibrio coralliilyticus* cells/mL	ANOVA or permutation ANOVA FDR-corrected p value (coral water compared to seawater)
1000	<0.001
100	**<0.001**
10	**0.063**

Coral Species: *Porites cylindrica*
Area: MPA
Temperature: 28 Celsius

Concentration of *Vibrio coralliilyticus* cells/mL	ANOVA or permutation ANOVA FDR-corrected p value (coral water compared to seawater)
1000	<0.001
100	<0.001
10	<0.001

Coral Species: *Acropora millepora*
Area: Fished Area
Temperature: 28 Celsius

Concentration of *Vibrio coralliilyticus* cells/mL	ANOVA or permutation ANOVA FDR-corrected p value (coral water compared to seawater)
1000000	0.014
100000	0.008
10000	**0.011**
1000	0.011
100	<0.001
10	<0.001

Coral Species: *Pocillopora damicornis*
Area: Fished Area
Temperature: 28 Celsius

Concentration of *Vibrio coralliilyticus* cells/mL	ANOVA or permutation ANOVA FDR-corrected p value (coral water compared to seawater)
1000	**<0.001**
100	0.002
10	**0.251**

Coral Species: *Porites cylindrica*
Area: Fished Area
Temperature: 28 Celsius

Concentration of *Vibrio coralliilyticus* cells/mL	ANOVA or permutation ANOVA FDR-corrected p value (coral water compared to seawater)
1000	<0.001
100	<0.001
10	<0.001

permutation p values are in bold

Table S2: PERMANOVA and PERMDISPERSION results for coral microbial community composition and dispersion

A. PERMANOVA pairwise tests

	t	p (permutation)
Porites cylindrica - Acropora millepora	14.69	0.001
Porites cylindrica - Pocillopora damicornis	20.12	0.001
Acropora millepora - Pocillopora damicornis	7.89	0.001

B. PERMDISPERSION averages

	average distance from centroid	standard error
Acropora millepora	36.79	2.07
Pocillopora damicornis	21.57	2.88
Porites cylindrica	12.86	1.32

C. PERMDISPERSION pairwise tests

	t	p (permutation)
Porites cylindrica - Acropora millepora	9.79	0.001
Porites cylindrica - Pocillopora damicornis	2.90	0.015
Acropora millepora - Pocillopora damicornis	4.38	0.002

Table S3: Coral microbial community composition

	Relative abundances (%)					
	Acropora millepora		*Pocillopora damicornis*		*Porites cylindrica*	
	MPA	Fished Area	MPA	Fished Area	MPA	Fished Area
Amoebophilaceae	0.01	0.01	0.55	0.70	2.50	4.87
Chroococcales	0.10	3.24	0.01	0.71	0.00	0.00
Rhodobacteraceae	0.17	0.18	0.35	2.28	0.64	0.68
Low abundance Endozoicimonaceae	3.31	7.32	0.41	5.19	3.83	0.90
Endozoicimonaceae 370251	17.17	23.11	1.63	5.21	0.10	0.07
Endozoicimonaceae 164076	2.25	2.11	0.51	0.69	0.12	0.05
Endozoicimonaceae 739464	0.03	0.04	0.02	0.02	1.02	1.16
Endozoicimonaceae 221108	2.78	1.43	0.10	0.13	0.25	0.22
Endozoicimonaceae 555869	4.33	3.80	0.99	1.17	85.28	82.75
Endozoicimonaceae 109431	0.10	0.16	24.67	21.22	0.12	0.06
Endozoicimonaceae 347784	30.10	22.76	60.13	53.62	0.86	0.08
Endozoicimonaceae 585094	3.54	2.83	0.02	0.03	0.58	0.60
Endozoicimonaceae new reference OTU101	31.81	25.53	0.20	0.18	0.08	0.07
Unassigned taxa	0.32	0.45	0.26	0.52	0.16	0.80
Low abundance archaea	0.28	0.28	0.51	0.18	0.06	0.13
Low abundance bacteria	3.69	6.74	9.63	8.16	4.39	7.56

Table S4. Relative abundance and analyses of Vibrionaceae for each coral species and site.

A. Percent relative abundance Vibrionaceae

	Acropora millepora		*Pocillopora damicornis*		*Porites cylindrica*	
	MPA	Fished Area	MPA	Fished Area	MPA	Fished Area
Mean	0.32	0.54	0.09	1.66	0.07	0.28
Standard error	0.08	0.21	0.04	1.35	0.03	0.21

B. ANOVA or permutation ANOVA p values for Vibrionaceae relative abundance for each coral species

	Acropora millepora	*Pocillopora damicornis*	*Porites cylindrica*
Origin	**0.311**	**0.032**	0.324
Village	**0.299**	**0.129**	0.385
Origin*Village	**0.317**	**0.059**	0.512

permutation p value are in bold

Table S5. Diversity of Vibrionaceae.

A. *Acropora millepora* Vibrionaceae OTU richness

	MPA	Fished Area
Mean	2.83	3.25
Standard error	0.48	0.81

B. *Acropora millepora* Vibrionaceae Shannon diversity

	MPA	Fished Area
Mean	0.43	0.38
Standard error	0.08	0.08

C. Permutation ANOVA p values for *Acropora millepora* OTU richness

Origin	0.725
Village	0.071
Origin*Village	0.341

D. Permutation ANOVA p values for *Acropora millepora* Shannon diversity

Origin	0.804
Village	0.566
Origin*Village	0.875
